# First-principles study of the T-phase monolayer MXenes Mo_2_N and Mo_2_NT_2_ (T = F, O) for anode application in lithium-ion batteries†

**DOI:** 10.1039/d5ra03426d

**Published:** 2025-05-28

**Authors:** Wenlong Xi, Patrick H.-L. Sit

**Affiliations:** a School of Energy and Environment, City University of Hong Kong Tat Chee Avenue, Kowloon Hong Kong China patrick.h.sit@cityu.edu.hk; b City University of Hong Kong Shenzhen Research Institute Shenzhen 518057 China

## Abstract

The exploration of high-performance anode materials plays a pivotal role in advancing the development of lithium-ion batteries (LIBs) for various applications. In this study, we investigate the potential of the MXene materials, T-phase Mo_2_N and Mo_2_NT_2_ (T = F, O) as anode materials for LIBs through the application of first-principles calculations. The results show that the diffusion rate of Li atoms on Mo_2_N is faster than that on Mo_2_NF_2_ and Mo_2_NO_2_, and the adsorption of a high concentration of Li atoms results in destruction of the surface structure of Mo_2_NF_2_. The calculated theoretical capacities for Mo_2_N and Mo_2_NO_2_ are determined to be 260.3 mA h g^−1^ and 225.3 mA h g^−1^, respectively, and the mean open-circuit voltages are computed to be 0.97 V and 0.73 V, respectively. Our results show that Mo_2_N and Mo_2_NO_2_ exhibit significant promise for utilization as anode materials in LIBs.

## Abstract 2

The exploration of high-performance anode materials plays a pivotal role in advancing the development of lithium-ion batteries (LIBs) for various applications.

## Graphical abstract

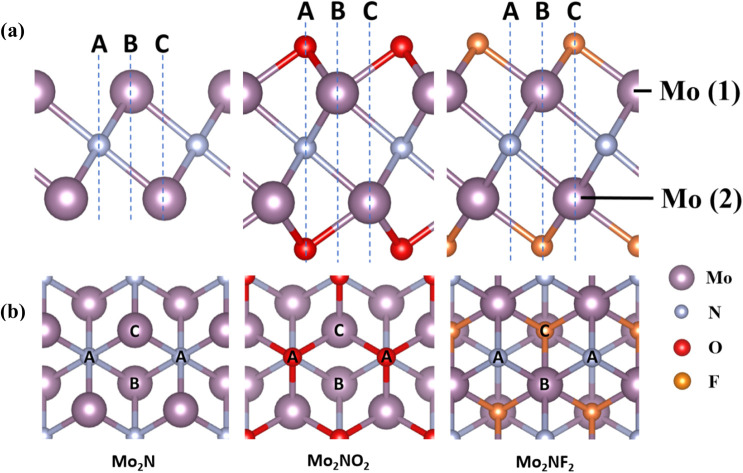

## 1 Introduction

With growing energy demand and increasingly prominent environmental problems, lithium-ion batteries (LIBs), as an efficient and reliable technology for energy storage, have become an important component in applications such as electric vehicles, renewable energy and portable electronic devices.^1–3^ However, in order to meet the increasing demand, it is still an urgent task to improve the lithium-ion battery performance in terms of the efficiency, production costs, energy density, cycle life, and safety,^4–7^ which require the search for novel battery materials. As a key component in LIBs, selection of negative electrode materials (anode) has an important impact on the battery performance.^8^ However, graphite anodes exhibit slow lithium-ion diffusion kinetics, leading to poor rate performance, and are prone to lithium plating at high charging rates, which poses safety risks.^9,10^ As a result, researchers are constantly looking for novel anode materials to improve battery performance.

The past decades have witnessed significant advancements in the exploration and development of two-dimensional (2D) nanomaterials within the scientific research communities. Among these emerging 2D nanomaterials, notable examples include graphene,^11–13^ transition metal oxides (TMOs),^14–16^ transition metal sulfides (TMDs),^17,18^ silicene,^19^ phosphorene^20^ and MXene^21,22^ have shown their promising potentials as negative electrode materials for LIBs. These 2D materials not only have high theoretical specific capacity, but also have the characteristics of excellent conductivity, reasonable open circuit voltage, structural stability and low diffusion energy barrier, this contributes to enhancing the power performance and cyclic stability of battery systems. Among them, MXene, as a new class of 2D transition metal carbon/nitrogen compounds, has attracted widespread attention in batteries research area due to its excellent electrochemical performance.^21^ Some MXenes, like Ti_2_C,^23^ Ti_3_C_2_,^24,25^ Mo_2_C,^26^ Nb_2_C,^27^ V_2_C,^27^ Ti_2_N^28^ and V_2_N,^29^ not only have high electronic and ionic conductivity, but also can regulate its properties through inclusion of surface functional groups, thereby changing its performance in LIBs.

As a new member of the MXene family, Mo_2_N exhibits high electrical conductivity, stable and MXene structure.^30,31^ Theoretically, Mo_2_N MXene exists in both the T and H phases, with density functional theory (DFT) calculations indicating that the H phase exhibits higher stability compared to the T phase.^32^ In the previous work, Mehta *et al.* found the H-phase Mo_2_N monolayer has low diffusion barrier and the high specific charge capacity with Li-adsorption indicate easy diffusion.^32^ Mehta *et al.* systematically studied the electrochemical properties of the T-phase Mo_2_N based on density functional theory (DFT) calculations and showed the metallic properties of Na-adsorbed Mo_2_N monolayers, which suggests that the material is promising to be used as anode materials for sodium-ion batteries.^33^ Yang *et al.* fabricated the composite materials, MoS_2_/T-Mo_2_N, with nitrogen doped carbon, and the results shown that it had high electrical conductivity and showed a good electrochemical performance with excellent cycling stability and high specific capacity.^34^

These results suggest that the Mo_2_N-based MXenes are promising anode materials in LIBs. However, it is worth noting that the surfaces of MXenes are usually covered with surface groups. In particular, it was suggested that the two Mo layers are generally covered with F or O functional groups, instead of bare Mo_2_N.^28,35,36^ In order to further allow us to further improve the properties and performance of the Mo_2_N-based MXenes as anodes *via* surface group modification, it is crucial to thoroughly investigate the lithium-ion storage capacity of these materials. This involves not only assessing their electrochemical performance but also understanding how structural features and surface characteristics influence Li ion uptake. A detailed analysis of the role played by functional groups in the lithium adsorption process is essential, as these groups can significantly affect the material's reactivity and ion transport mechanisms.

Therefore, in this work, the physiochemical properties of the T-phase Mo_2_N and Mo_2_NT_2_ (T = O, F) two-dimensional nanosheets as new anode materials for LIBs are systematically studied through first principles calculations. The diffusion energy profiles of the Li atoms indicate that the Li atoms have low energy barriers on the Mo_2_N and Mo_2_NT_2_ surfaces, which potentially allows fast charge–discharge cycle. Furthermore, according to their voltage curves and storage capacities, we found that both Mo_2_N and Mo_2_NO_2_ have suitable storage capacities and low open circuit voltages. However, the adsorption of high concentration of Li atoms leads to destruction of the surface structure of Mo_2_NF_2_. Therefore, Mo_2_N and Mo_2_NO_2_ were found to be very promising for high performance LIBs electrodes, and can attract further attention from experimenters.

## 2 Computational method

In the present study, first-principles calculations based on density functional theory (DFT) were performed using the plane-wave self-consistent field (PWscf) module of the Quantum ESPRESSO computational package.^37,38^ The UltraSoft pseudopotentials (USPP) were used,^39^ for their ability to accurately describe the core-electron interactions while reducing computational costs. This was combined with the Perdew–Burke–Ernzerhof (PBE) exchange–correlation functional to perform the calculations.^40^ To effectively capture the van der Waals (vdW) forces, which are critical in layered materials, we applied the Grimme's semiempirical DFT-D2 approach.^41,42^ The plane-wave basis set with the kinetic energy cutoffs of 60 Ry for the wavefunctions and 480 Ry for the augmented charge density. The Monkhorst–Pack grid of 6 × 6 × 3 *k*-points was adopted to sample the Brillouin zone. For the MXene structure, we considered a monolayer 2 × 2 × 1 periodic supercell. To prevent interactions between layers in the *z* direction, a vacuum space larger than 15 Å was introduced. The diffusion energy barrier and minimum energy path of the Li atoms on monolayers of Mo_2_N and Mo_2_NT_2_ were calculated using the nudge elastic band (NEB) method.^43^ To investigate the charge transfer mechanisms and electronic redistribution characteristics, the Bader charge analysis method was employed.^44,45^

## 3 Results and discussion

### 3.1 Structures and electronic properties of Mo_2_N and Mo_2_NT_2_

**Fig. 1:**
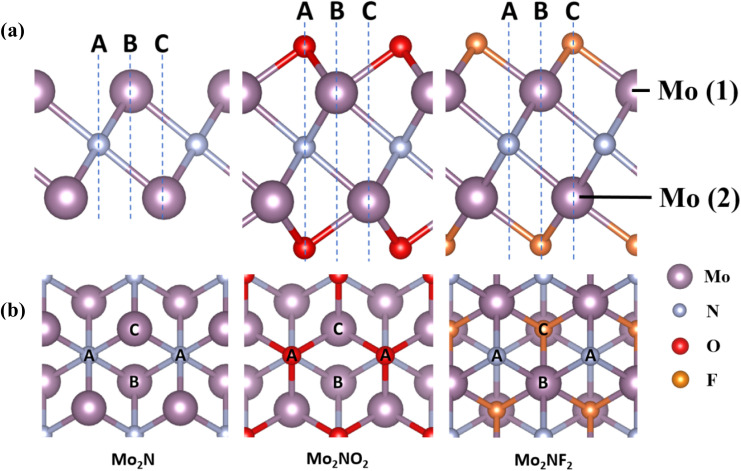
(a) Side view and (b) top view of the structural model of Mo_2_N and Mo_2_NT_2_ (T = O, F). Mo is purple, N is light blue, O is red, and F is orange. A, B, and C are the three different adsorption sites of the Li atom on Mo_2_N and Mo_2_NT_2_.

Fig. 1a and b show the side view and top view of optimized Mo_2_N and Mo_2_NT_2_ (T = O, F) structures. In the Mo_2_N structure, in which a single layer of nitrogen atoms (N) is sandwiched between the upper and lower layers of Mo atoms. The two layers of Mo are denoted as Mo (1) and Mo (2) respectively. Six Mo atoms are coordinated to one N atom to form an octahedral structure. In the layered Mo_2_NO_2_ and Mo_2_NF_2_, the Mo atoms are bonded to the surface O atoms and F atoms, respectively. Previous studies^46–48^ of MXenes have shown that functional group atoms, O and F, are more likely to occupy hollow sites (Site A/C) instead of the top position (Site B) as shown in Fig. S1.† As a result, the vacant Site A and Site C, which are positioned straightly on the top of N atoms and the Mo (2) atoms in the monolayer Mo_2_N, are identified as possible locations for the O/F functional group atoms. In Configuration I shown in Fig. S1,† the O/F atoms are situated at the C sites on either side of the monolayer Mo_2_N, whereas in Configuration II, the O/F atoms occupy the A sites on both sides of Mo_2_N. We then calculated the total energy of each configuration and found that Mo_2_NO_2_ of Configuration II and Mo_2_NF_2_ of configuration I exhibited the lowest energy and were considered to be the most stable conditions (Table S1†).

After structural optimization, we obtained the lattice parameters *a* of Mo_2_N as 2.79 Å in Table S2,† these values align closely with findings from prior experimental and theoretical investigations, ranging between 2.78 Å and 2.89 Å.^30,33,49,50^ The lattice parameters *a* of Mo_2_NO_2_ and Mo_2_NF_2_ are 2.88 Å and 2.78 Å, respectively. Due to the symmetry of Mo_2_N and Mo_2_NT_2_, the three different sites of adsorption of the Li atom on Mo_2_N and Mo_2_NT_2_ correspond to A, B, and C as depicted in Fig. S2.† These sites also align vertically to the nitrogen atoms, upper Mo (1) atoms, and lower Mo (2) atoms, respectively.

**Fig. 2:**
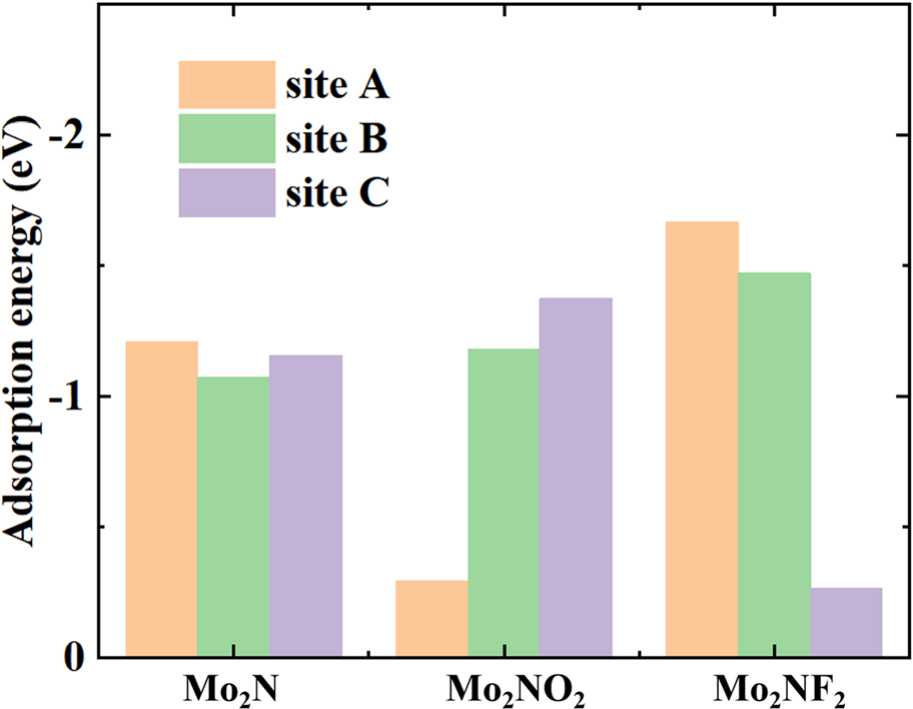
Calculated adsorption energies of Li at different adsorption sites on monolayers of Mo_2_N and Mo_2_NT_2_ (T = F, O).

We then examined the favorable adsorption locations for Li atoms. The adsorption energies at different sites are illustrated in Fig. 2. The negative value represents the effective adsorption, and with the more negative value of *E*_a_ signifying greater stability for the Li atoms to be adsorbed on Mo_2_N and Mo_2_NT_2_. As illustrated in Fig. 2, detailed analysis of the adsorption energetics reveals that the A site represents is most energetically favorable site for lithium adsorption in both Mo_2_N and Mo_2_NF_2_, whereas the C site demonstrates highest stability in the case of Mo_2_NO_2_. The adsorption energies on Mo_2_N, Mo_2_NO_2_ and Mo_2_NF_2_ are −1.21, −1.37 and −1.67 eV, respectively. The modification of the surface with the O atoms and F atoms effectively improves the adsorption of Li atoms on Mo_2_NO_2_ and Mo_2_NF_2_. For Mo_2_N, the difference of adsorption energy among each site is small, only about 0.14 eV, and the adsorption energy of the B site is less negative than of the C site. On the contrary, the difference in adsorption energy between the A site and the C site on Mo_2_NT_2_ is much larger on Mo_2_N.

**Fig. 3:**
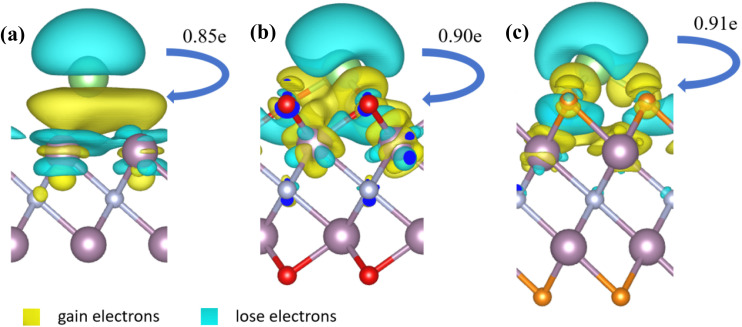
Charge density difference (CDD) associated with the (a) Mo_2_N (b) Mo_2_NO_2_ and (c) Mo_2_NF_2_ monolayers after Li atom adsorption, with the yellow iso-surfaces indicating electron gain and blue iso-surfaces indicating electron loss.

In the Mo_2_N and Mo_2_NT_2_ (T = F, O) structures, the Li atom is bound to three adjacent Mo atoms or T atoms, respectively. The average Li–Mo length was 2.75 Å, the average Li–O length was 1.90 Å, and the average Li–F length was 1.80 Å for the three cases as shown in Table S2.† Due to the modification of the surface with the F and O atoms, the coulombic interaction between Mo_2_NT_2_ and the Li atom is strengthened. To verify this and further understanding of Li atom adsorption behavior on monolayers, we performed detailed Bader charge analysis coupled with charge density difference (CDD) calculations. The CDD results in Fig. 3 demonstrate that following the incorporation of a Li atom through adsorption, the charge density around the monolayers and the Li atom is redistributed, and the electrons above the Li atom are transferred to the region between the monolayers and the Li atom. The Bader charge analysis in Table 1 shows that the adsorbed Li atom transfers 0.85, 0.90, or 0.91 electrons to the monolayer Mo_2_N, Mo_2_NO_2_, or Mo_2_NF_2_, respectively. Therefore, the adsorption of Li on the surface of Mo_2_NT_2_ (T = F, O) is stronger than that on the surface of Mo_2_N due to the reduction of the bond length and the increase of charge transfer.

**Table 1:** Average charge of different elements in the Mo_2_N and Mo_2_NT_2_ monolayers from Bader charge analysis. Li-Mo_2_N, Li-Mo_2_NO_2_ and Li-Mo_2_NF_2_ denote the monolayers with a Li atom adsorbed

	Mo	N	O	F	Li
Mo_2_N	0.67	−1.34			
Li-Mo_2_N	0.55	−1.31			0.85
Mo_2_NO_2_	1.63	−1.39	−0.93		
Li-Mo_2_NO_2_	1.59	−1.39	−1.01		0.90
Mo_2_NF_2_	1.34	−1.38		−0.65	
Li-Mo_2_NF_2_	1.27	−1.36		−0.70	0.91

**Fig. 4:**
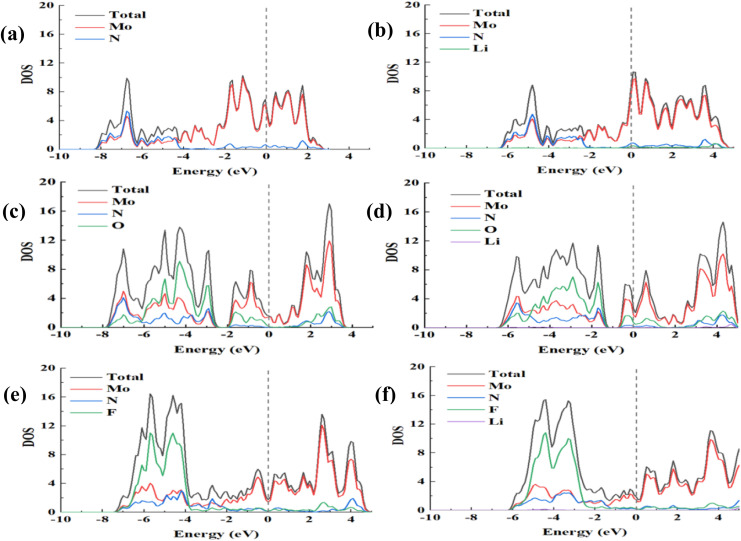
Total density of states (TDOS) and projected density of states (PDOS) for (a) Mo_2_N, (b) Li-adsorbed Mo_2_N, (c) Mo_2_NO_2_, (d) Li-adsorbed Mo_2_NO_2_, (e) Mo_2_NF_2_ and (f) Li-adsorbed Mo_2_NF_2_.

After adsorbing the Li, whether the monolayer becomes a conductor or an insulator is crucial to its performance as a promising anode material for LIBs. To this end, we studied the total density of states (TDOS) and projected density of states (PDOS) of the monolayer Mo_2_N, Mo_2_NO_2_, and Mo_2_NF_2_ before and after Li adsorption as shown in Fig. 4. We can see that the metallic properties of Mo_2_NT_2_ do not change after O and F atom modification of the Mo_2_N surface. Moreover, after Li adsorption, Mo_2_N, Mo_2_NO_2_ and Mo_2_NF_2_ still remain the metallic properties. The metallic properties mainly come from the orbitals of the Mo as their peaks are prominent close to the Fermi level. The DOS at the Fermi level of Mo_2_N and Mo_2_NO_2_ are stronger than Mo_2_NF_2_ after Li adsorption. The metallic property of Mo_2_N, Mo_2_NO_2_, and Mo_2_NF_2_ makes them the potential candidates as electrode materials for LIBs.

### 3.2 Li atoms diffusion barrier

**Fig. 5:**
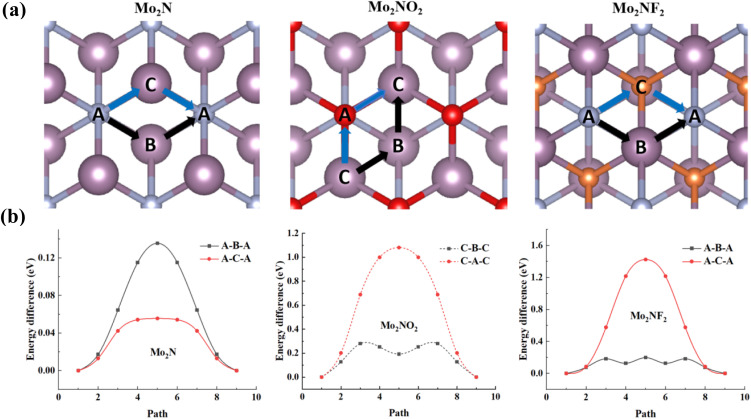
Lithium diffusion characteristics: (a) schematic representation of minimum energy pathways and (b) corresponding activation energy barriers for Li migration on Mo_2_N, Mo_2_NO_2_ and Mo_2_NF_2_ surfaces.

Subsequently, we investigated the diffusion behavior of a lithium atom on the surfaces of Mo_2_N, Mo_2_NF_2_, and Mo_2_NO_2_. The configuration of the energetically preferred adsorption sites for a Li atom on Mo_2_N, Mo_2_NF_2_, and Mo_2_NO_2_, which we identified in the previous section, was used as the initial state. As shown in Fig. 5a, based on the symmetry of the Mo_2_N and Mo_2_NF_2_ monolayers, we considered two possible migration paths, ABA/ACA or CAC/CBC. The ABA path denotes that the Li atom travels from the initial the A site to the B site, and then to an adjacent A site; the ACA path represents the Li atom traveling from the initial A site to the C site and to an adjacent A site. In Fig. 5a, the ABA and ACA pathways are represented by black and blue arrows, respectively. For the Mo_2_NO_2_ monolayer, we also considered two possible migration paths similarly, CAC and CBC pathways, which are also represented by black and blue arrows, respectively.

Fig. 5b illustrates the computed diffusion energy barrier lines along with the associated migration pathways. Our findings indicate that the energy barrier for the ABA configuration of Mo_2_N is higher than that of the ACA configuration, whereas the ABA configuration of Mo_2_NF_2_ has a significantly lower energy barrier compared to the ACA configuration. In the case of Mo_2_NO_2_, the energy barrier for the CAC configuration exceeds that of the CBC configuration. The minimum energy barriers for Li diffusion are 0.05, 0.20, and 0.19 eV for the Mo_2_N, Mo_2_NF_2_, and Mo_2_NO_2_ monolayers, respectively. This indicates that the migration of Li atoms on Mo_2_N occurs at a significantly higher rate compared to that on Mo_2_NF_2_ and Mo_2_NO_2_. However, the calculated diffusion barriers on the Mo_2_N, Mo_2_NF_2_, and Mo_2_NO_2_ monolayer structures are lower to that of alternative anode materials, including MoN_2_ (0.78 eV),^51^ graphene (0.33 eV),^52^ phosphorene (0.76 eV),^53^ silicene (0.23 eV),^19^ graphite (0.5–1.2 eV)^54^ and Ti_2_NO_2_ (0.25 eV).^28^ The lower energy barriers indicate that they are suitable for application as anode materials for LIBs. Since the order of lithium-ion diffusion barrier is Mo_2_NF_2_ > Mo_2_NO_2_ > Mo_2_N, the Li migrates most easily on the surface of the exposed Mo_2_N monolayer, while the presence of the F and O surface groups weakens the migration of the Li. However, in comparison with conventional electrode materials, the remarkably low lithium diffusion energy barrier exhibited by the nitride MXene indicates that it allows high Li mobility. This is anticipated to enhance cycling performance and increase discharge/charge rates when utilized as anode materials in LIBs.^55^

### 3.3 Open circuit voltage and theoretical capacity

From the application point of view, the open circuit voltage (OCV) and Li storage theoretical capacity of the electrode material are important characteristics. Here, we first studied the adsorption of different concentrations of the Li atoms on the monolayer Mo_2_N, Mo_2_NF_2_ and Mo_2_NO_2_. To assess the theoretical maximum adsorption capacity, we maintained the use of a 2 × 2 × 1 supercell and increased the number of Li atoms adsorbed on each side of the electrode material. In this context, we considered the Li/Li+ half-cell reaction as:^28^(Mo_2_N/Mo_2_NT_2_ + *x*Li + *x*e^−^ ↔ Li_*x*_Mo_2_N/Li_*x*_Mo_2_NT_2_)

We first calculated the average adsorption energy (*E*_a_) associated with each Li atom as a function of the Li atom concentration. The average adsorption energy of each concentration of the Li atoms on Mo_2_N/Mo_2_NT_2_ is calculated using eqn (1):

1 *E*_a_ = (*E*_Mo_2_N/Mo_2_NT_2__ + *xE*_Li_ − *E*_Li_*x*_Mo_2_N/Li_*x*_Mo_2_NT_2__)/*x*

where *E*_Mo_2_N/Mo_2_NT_2__ is the energy of the Mo_2_N/Mo_2_NT_2_, *E*_Li_*x*_Mo_2_N/Li_*x*_Mo_2_NT_2__ is the energy of Li_*x*_Mo_2_N/Li_*x*_Mo_2_NT_2_, E_Li_ represents the energy of a Li atom within a body centered cubic crystal structure, while *x* denotes the concentration of Li atoms.

**Fig. 6:**
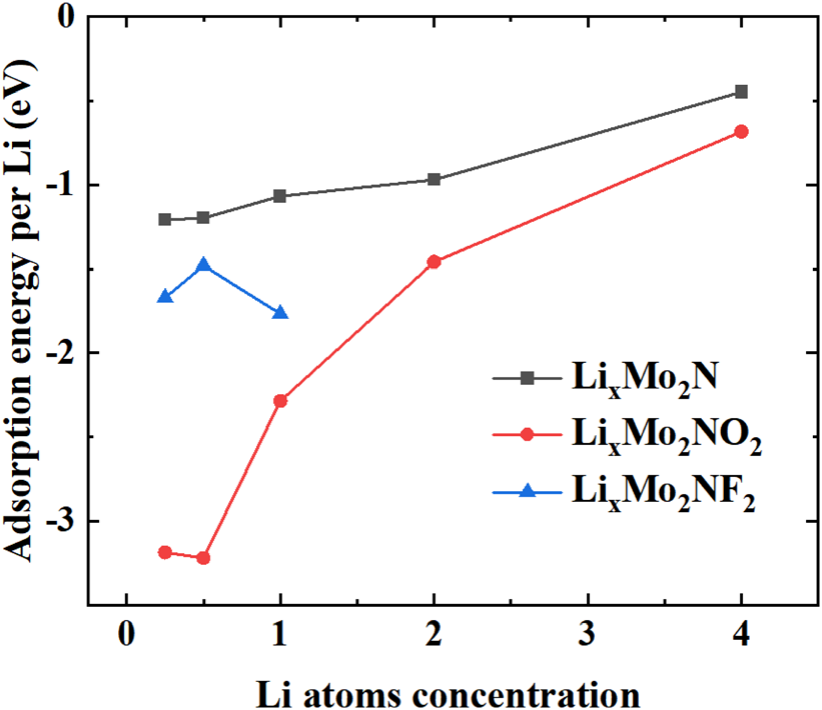
Average adsorption energies (*E*_a_) at different Li atom concentration.

We investigated various adsorption configurations by varying the number of Li atoms adsorbed on two-dimensional monolayers, represented by the formula Li_*x*_Mo_2_N, where *x* takes the values of 0.25, 0.5, 1, 2, and 4. As illustrated in Fig. 6, an increase in *x* corresponds to more Li atoms coverage on the Mo_2_N monolayer. For *x* = 2, both sides of the Mo_2_N layer are fully saturated with one layer Li atoms, while *x* = 4 indicates a double-layer of Li atoms. Thus, a higher *x* value suggests enhanced storage capacity through multilayer adsorption. Fig. 6 shows the *E*_a_ of Li atoms as a function of *x*. It can be seen that as the quantity of adsorbed lithium atoms rises, *E*_a_ gradually becomes less negative due to the enhancement of repulsive force between metal ions. When *x* ≥ 0.5, the structure of Mo_2_NF_2_ is destroyed due to bond formation of the Li atoms with the F atoms, which leads to the detachment of F from the Mo_2_NF_2_ surface, as depicted in Fig. S3.† This suggests that the maximum value of *x* for Mo_2_NF_2_ is not larger than 0.5. Through Bader charge analysis, we found that the charge of F and Mo are −0.65*e* and 1.64*e*, respectively in Mo_2_NF_2_. The charge of O and Mo are −0.98*e* and 1.34*e* in Mo_2_NO_2_, respectively. The F groups react with Li ions to generate the by-product LiF was also reported previously.^56^ Based on these results, we considered next only the OCV and capacity of the Mo_2_N and Mo_2_NO_2_ monolayers.

The OCV is a key aspect to consider when evaluating the performance of LIBs. While charging and discharging, the OCV can be assessed through the variation in the total energy difference before and after the adsorption of different number of metallic Li atoms. For the OCV involving *x*Li atoms intercalation reactions, the following calculation formula for the energy difference can be used:^57^2
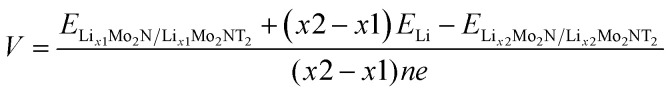
where *E*_Li_*x*_Mo_2_N/Li_*x*_Mo_2_NT_2__is the energy of Li_*x*_Mo_2_N/Li_*x*_Mo_2_NT_2_, *E*_Li_ represents the cohesive energy per lithium atom in its body-centered cubic (bcc) metallic crystal structure, which serves as a reference state for electrochemical potential calculations, *x* is the concentration of the Li atoms, *n* represent valence state of the adsorbed metal Li atoms (*n* = 1) and *e* represents elementary charge of single electron.

**Fig. 7:**
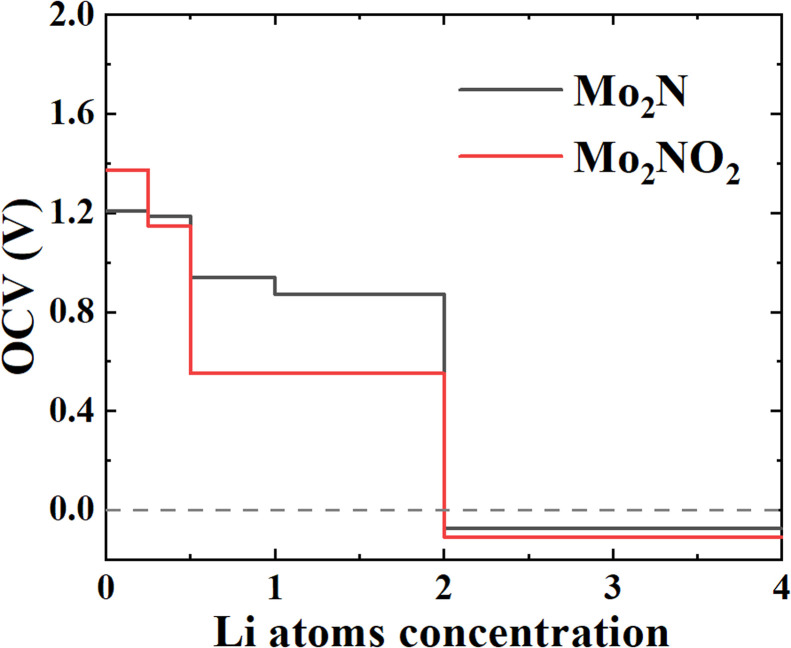
The OCV at different Li atoms concentration adsorbed on the Mo_2_N/Mo_2_NO_2_ monolayers.

Fig. 7 shows the OCV at different Li atoms concentration adsorbed on the Mo_2_N/Mo_2_NO_2_ monolayers. As shown in the figure, when the Li concentration approaches 2 (full monolayer coverage), the OCV becomes negative for both Mo_2_N and Mo_2_NO_2_. This indicates that it is not favorable for Li atoms to further adsorb on Mo_2_N and Mo_2_NO_2_ compared to the atoms in the Li metal electrode, suggesting that the maximum Li concentration is when *x* = 2 for both Mo_2_N and Mo_2_NO_2_. Some previous works also adopted this criterion to determine the Li storage limit.^57–59^ According to eqn (2), the average OCV of the monolayer Mo_2_N and Mo_2_NO_2_ when used as anode materials in LIBs are 0.97 V and 0.73 V respectively. The comparison of the voltages with other simulation works on MXene, *e.g.* Ti_2_N (0.67 V)^28^ and Ti_2_NO_2_ (1.00 V)^28^ are also closed with our simulation results. And these values of Mo_2_N and Mo_2_NO_2_ were found to be both positive and in the range of 0–1 V. The feasibility of Mo_2_N, Mo_2_NO_2_ monolayer structures as advanced anode materials for rechargeable Li-ion batteries is further demonstrated, and the O groups modified Mo_2_N surface (Mo_2_NO_2_) decreases the average OCV.

From the maximum Li concentration above, the theoretical specific capacity of the of Mo_2_N and Mo_2_NO_2_ battery can be determined according to eqn (3):3
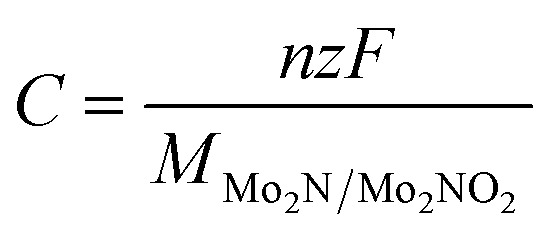
where, *z* = 1 for Li, *x* is the number of Li atoms adsorbed, *F* is the Faraday constant (26 801 mA h mol^−1^), and M_Mo_2_N/Mo_2_NO_2__ is the molar weight Mo_2_N or Mo_2_NO_2_.

The theoretical capacities of Mo_2_N and Mo_2_NO_2_ as anode materials for Li ion batteries were calculated to be 260.3 mA h g^−1^ and 225.3 mA h g^−1^, respectively. We find there are experimental papers on other MXene like V_2_CT_*x*_ (260 mA h g^−1^),^27^ Nb_2_CT_*x*_ (170 mA h g^−1^),^60^ Ti_3_C_2_*T*_*x*_ (330 mA h g^−1^),^61^ which show similar capacities to those in our calculations. And they are higher than some other reported electrode materials, such as TiO_2_ (200 mA h g^−1^)^62^ and Mn_2_CF_2_ (167 mA h g^−1^).^63^

## 4 Conclusions

In summary, we investigated in this work the potential of the novel MXene materials, Mo_2_N and Mo_2_NT_2_ (T = F, O) as the anode materials for lithium-ion batteries using density functional theory (DFT) calculations. The Mo_2_N, Mo_2_NO_2_, and Mo_2_NF_2_ was found to maintain their metallic properties before and after adsorption of Li atoms. The Mo_2_N, Mo_2_NF_2_, and Mo_2_NO_2_ monolayers also show low energy barriers for ionic diffusion, which are 0.05, 0.20, and 0.19 eV, respectively. Among them, the energy barrier of ionic diffusion on the surface of Mo_2_N is the lowest, indicating that it has a faster diffusion rate in LIBs. When a high concentration of Li atoms is adsorbed, the Li bonds with the F to form LiF, causing the F to detach from the Mo_2_NF_2_ surface and destroy its structure. In addition, the theoretical capacities of Mo_2_N and Mo_2_NO_2_ are 260.3 mA h g^−1^ and 225.3 mA h g^−1^, respectively, and the mean open circuit voltages are calculated to be 0.97 V and 0.73 V, respectively. Our results indicate the possible usefulness of Mo_2_N, Mo_2_NO_2_ as anode materials for LIBs, while Mo_2_NF_2_ is not appropriate for use as anode materials in LIBs.

## Data availability

Data will be made available on request.

## Author contributions

WX was responsible for conducting the investigation, performing simulations, analyzing data, and drafting the manuscript. PHLS contributed to the discussions, provided supervision, originated the research idea, secured research resources, managed the project, oversaw the research activities, and engaged in the review and editing process.

## Conflicts of interest

The authors affirm that they have no competing interests to disclose.

## Supplementary Material

RA-015-D5RA03426D-s001
