# Supplementary material for: First-principles study of the T-phase monolayer MXenes Mo2N and Mo2NT2 (T = F, O) for anode application in lithium-ion batteries
Source: RSC Adv. 2025 May 28;15(22):17795–802. doi: 10.1039/d5ra03426d (PMC12117402; doi:10.1039/d5ra03426d)
Supplement: RA-015-D5RA03426D-s001 [file RA-015-D5RA03426D-s001.pdf]

Supplementary Information (SI) for RSC Advances.
This journal is © The Royal Society of Chemistry 2025

Supporting Information

First-principles study of the T-phase monolayer MXene Mo_2N and Mo_2NT_2 (T = F, O) for anode application in lithium-ion batteries

Wenlong Xi [a], Patrick H.-L. Sit [a,*]

[a] School of Energy and Environment, City University of Hong Kong, Tat Chee Avenue, Kowloon, Hong Kong, China

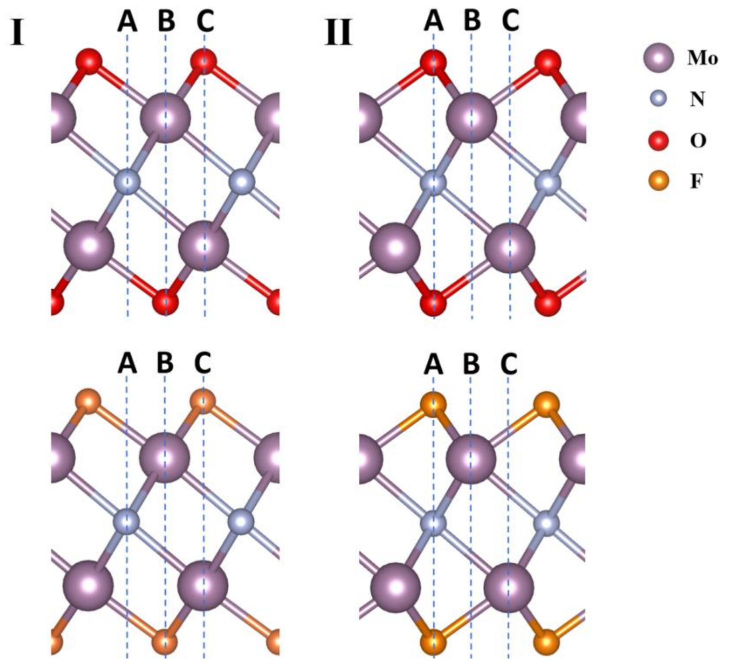

Fig. S1 Tow possible configurations of O or F atom locations of Mo_2NO_2 and Mo_2NF_2.

Table S1 The relative energy (eV) of each configuration of Mo_2NO_2 and Mo_2NF_2.

	I	II
Mo_2NO_2	2.72	0
Mo_2NF_2	0	2.73

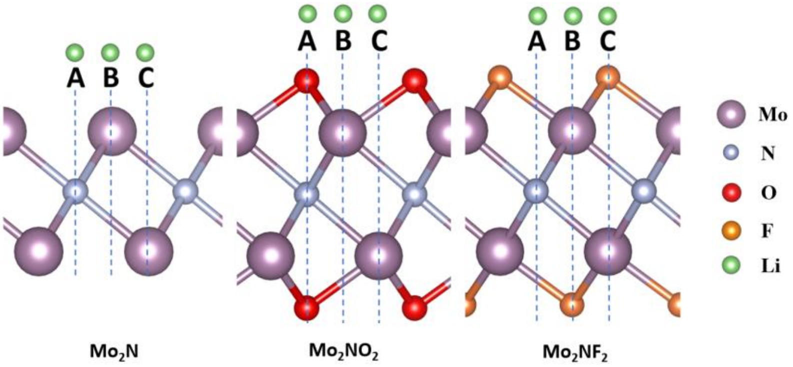

Fig. S2 Three different adsorption sites of the Li atom on Mo_2N and Mo_2NT_2.

Table S2: Mo_2N and Mo_2NT_2 optimized lattice parameters (a, b, c), and average atom distances.

	a(Å)	b(Å)	c(Å)	d_{Li-Mo} (Å)	d_{Li-O} (Å)	d_{Li-F} (Å)
Mo_2N	2.79	2.79	2.85	2.75	-	-
Mo_2NO_2	2.88	2.88	5.23	-	1.90	-
Mo_2NF_2	2.78	2.78	5.97	-	-	1.80

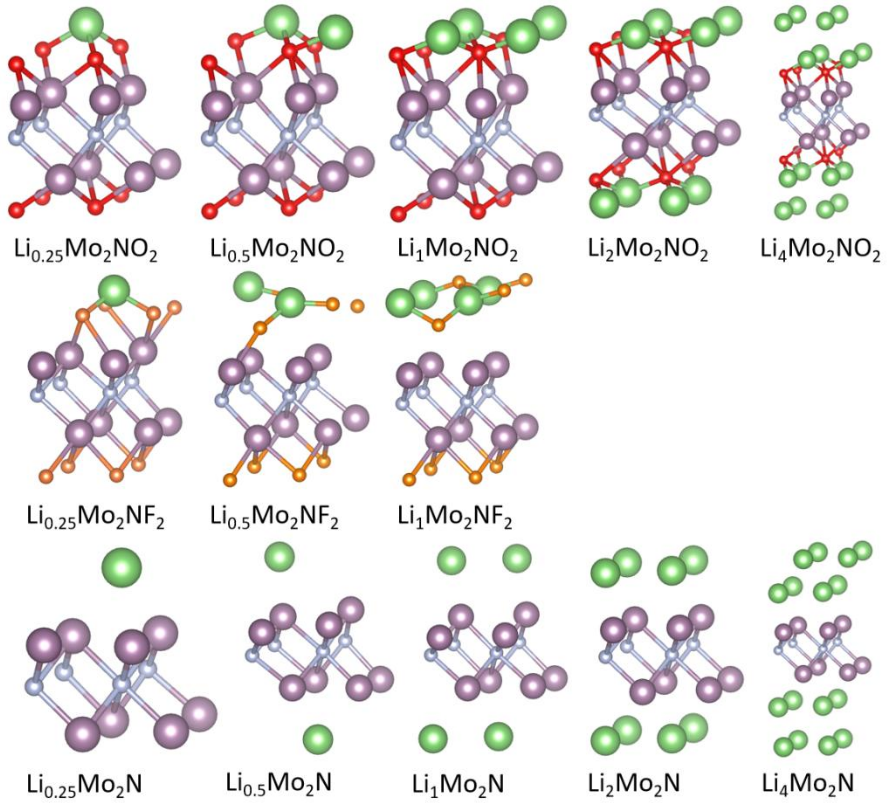

Fig. S3 Adsorption configurations with different Li concentrations.
